# Color Characteristic Alteration of Different Yttrium Oxide-Containing Multilayer Partially Stabilized Zirconia at Different Sintering Rates

**DOI:** 10.1055/s-0045-1806929

**Published:** 2025-04-23

**Authors:** Atthasit Boonbanyen, Niwut Juntavee, Apa Juntavee

**Affiliations:** 1 Division of Biomaterials and Prosthodontics Research, Faculty of Dentistry, Khon Kaen University, Khon Kaen, Thailand; 2 Department of Prosthodontics, Faculty of Dentistry, Khon Kaen University, Khon Kaen, Thailand; 3 Division of Pediatric Dentistry, Department of Preventive Dentistry, Faculty of Dentistry, Khon Kaen University, Khon Kaen, Thailand

**Keywords:** color appearance, contrast, sintering rate, translucency, opalescence, zirconia

## Abstract

**Objectives:**

Sintering influences the optical properties of zirconia. This study examined the effect of altering sintering rates on color characteristics of 3, 4, and 5 mol% yttria (Y)-containing multilayer zirconia.

**Materials and Methods:**

A total of 135 specimens (width × length × thickness = 11.2 × 20 × 1.5 mm) were prepared from multilayer (cervical [C], middle [M], and incisal [I]) 3Y, 4Y, and 5Y zirconia, and randomly sintered at regular (RS: 10 °C/min), fast (FS: 35 °C/min), and speed (SS: 70 °C/min) sintering ( *n* = 15/group). Translucency parameter (TP _00_ ), contrast ratio (CR), opalescence parameter (OP), and color difference (∆E _00_ ) were evaluated with the CIEL*a*b* system. Microstructure, crystalline (monoclinic [m], tetragonal [t], and cubic [c]) phases, and surface roughness (Ra) were evaluated by scanning electron microscopy (SEM), X-ray diffraction (XRD), and 3D-digital topography.

**Statistical Analysis:**

ANOVA and Bonferroni comparisons were determined for significant differences ( *p <* 0.05).

**Results:**

Significant differences in TP _00_ , CR, OP, and ∆E _00_ of zirconia types, layers, sintering rates, and their interactions were indicated ( *p <* 0.05). Significant increases in TP _00_ and ∆E _00_ , but decreases in CR and OP, upon rising the amount of Y (5Y > 4Y > 3Y), region (I > M > C), and speed sintering (SS > FS > RS) ( *p <* 0.05) were observed. Nevertheless, the color alteration was within an acceptable threshold (∆E _00_ ≤ 1.8); Ra values: 3Y > 4Y > 5Y. SEM indicated a larger grain for 5Y > 4Y > 3Y. XRD indicated higher t-phase in 3Y, whereas higher c-phase in 5Y.

**Conclusion:**

Increasing translucency and color alteration, and decreasing contrast and opalescence were influenced by Y content (5Y > 4Y > 3Y), region (I > M > C), and sintering rate (SS > FS > RS). Nonetheless, color alterations were within acceptable limits, suggesting the speed sintering rate to produce better color characteristics of restoration.

## Introduction

Progressively increasing demand for aesthetic restorations has been steering the development of several new ceramic materials to provide restorations with life-like appearance. The glass-containing ceramic was developed for restoring the severely damage teeth in the esthetic zone on account of their high translucent characteristics. However, the foremost weakness of glass ceramics is their low toughness and strength, which are prone to fracture during mastication, thus limiting their use only for the fabrication of partial coverage restorations, full anatomical crowns, and short-span anterior bridges. The zirconia dental ceramics were technologically advanced to overcome the less toughness and strength of the glass ceramics, which could fabricate long-span restoration, in either anterior or posterior regions. 1 Usually, dental zirconia presents in three distinctive crystalline phases depending on different ambient temperatures. The monoclinic ( *m* ) phase is the only form that remains constant at room temperature. Once heating zirconia to 1,170 °C, the tetragonal ( *t* ) phase can develop. Ultimately, the cubic ( *c* ) phase appears as the temperature increases up to 2,370 °C. On the contrary, the zirconia phases are reversed from *c* -phase and *t* -phase to *m* -phase as it cools down from high temperature to room temperature. To stabilize the *t* - and *c* -phases at room temperature, the yttrium oxide (Y _2_ O _3_ ) dopant was usually added into zirconia during the manufacturing process. The yttria content of yttria-doped zirconia usually ranges from 3 to 5 mol%. The 3 mol% yttria-stabilized tetragonal zirconia polycrystal (3Y-TZP) was first developed, predominantly consisting of the *t* -phase, which exhibited an extreme strength from the phase transformation ( *t* → *m* phase) toughening mechanism, led to an approximately 4 to 5% volume expansion of the zirconia grain, and generated the compressive force at the crack regions as a consequence. 2 However, its optical characteristics, including the dull-white appearance and high opacity, are the drawbacks of having a small grain size, large grain boundaries, and high amounts of aluminum oxides. Thus, it is used as a substructure to be veneered with glass ceramic to simulate the natural tooth appearance. Yet, the delamination of glass ceramic from the zirconia substrate is a major disadvantage. Consequently, the monolithic 3Y-TZP showed superior fracture resistance and better translucency by refining the smaller grain size than the prototype as well as decreasing the alumina contents, which led to reduction in the scattering of the incidence light of the alumina dopants, located between the zirconia grain boundaries. Still, it possesses low translucency, which is not suitable for restorations in the aesthetic zone. 3 Extremely translucent zirconia is contemporarily industrialized by adding 4 to 5 mol% of Y _2_ O _3_ to produce the 4 to 5 mol% partially stabilized zirconia (4Y-PSZ and 5Y-PSZ), which comprises more *c* - phases depending on the amount of yttria stabilizer. Both 4Y-PSZ and 5Y-PSZ provide significantly higher translucency than 3Y-TZP because the isotropic structure of the *c* -phase dominates the light refraction to be in a straighter line than the asymmetrical structure of the *t* -phase. Likewise, they provide a larger grain size with lower grain boundaries, which evokes less light scattering. The modification of the sintering process, for example, increasing the sintering temperature and prolonging the sintering time, has been reported as a suitable sintering protocol for enhancing translucency of the monolithic zirconia. 4 5 Recently, the multilayer zirconia was introduced comprising incisal (I), middle (M), and cervical (C) layers to simplify mimicking the color gradient found in the natural tooth appearance, which dramatically benefits clinicians in the fabrication of dental restorations. 6

Color characteristics, comprising translucency, contrast, opalescence, and color perception, are the prime concerns in the selection of ceramics for fabrication of aesthetic restoration. 7 8 9 Translucency is described as the amount of light transmitted through a material, and it was considered in terms of translucency parameter (TP _00_ ) and contrast ratio (CR). 10 A highly translucent material would demonstrate a greater TP _00_ value, but a lower CR value since these parameters are adversely correlated. 11 The translucency of zirconia is associated with several factors, including the microscopic structure and grain size, the chemical composition, the phase composition, and the external surface topography. 12 13 14 15 The low translucency of zirconia makes it more effective in concealing the color of the underlying substrate. 16 17 The phenomenon of visible light scattering and transmitting on the substrate is related to the dimensions of grains, crystalline microstructures, coloring pigments, and porosities of the material. 18 The longer wavelengths (orange to red) can pass through the substrates, while the shorter wavelengths (purple to blue) scatter on the surface. The restoration would look blueish once the light is redirected from it and display an orange illusion as the light transmits throughout. This occurrence is recognized as opalescence and is considered in terms of opalescence parameter (OP), which creates the restoration closely imitating the life-like human enamel (OP ≈ 19.8–27.6). 10 19 Studies reported that the opalescence of zirconia could be increased by raising the concentration of metal oxides, such as ZrO _2_ , Y _2_ O _3_ , SnO _2_ , and V _2_ O _5_ , and by creating larger grain size than the visible wavelength. 20 21 Concerning color perception, color difference (∆E _00_ ) is used to determine the degree of perception of color alteration, which is based on perceptibility threshold (PT, ∆E _00_ = 0.81) and the acceptability threshold (AT, ∆E _00_ = 1.80). The value of ∆E _00_ <0.81 signified “ *clinically indifferent,* ”, ∆E _00_ = 0.81–1.80 signified “ *clinically acceptable,* ” and ∆E _00_ >1.80 signified “ *clinically unacceptable* ” perception of color difference. 7 17 The more the ∆E _00_ increases, the more the distinguishability as well as the less acceptability in color alteration.

Sintering is a vital process for the fabrication of zirconia restorations. This consists of four parameters: sintering rate, sintering temperature, sintered holding time, and cooling rate. The sintering rate is primarily the crucial parameter to generate heat per minute (°C/min) in the furnace until reaching the final sintering temperature. The sintering rates traditionally ranged between 5 and 20°C/min depending on the manufacturer's recommendation, which is a time-consuming and energy-consuming process. 22 Shortening the sintering time is beneficial not only to dental laboratories but also to chairside clinical practice in the efficient fabrication of zirconia restoration. 5 Several attempts were introduced to improve color characteristics of zirconia restoration through the manufacturing process, such as modifying microstructure, chemical elements, and relative phase distribution. Contrariwise, it is common to modify the sintering processes. 3 4 5 11 22 23 24 25 26 27 28 Predominantly, a shortened sintering time by increasing the sintering rate is an attractive method; however, the influence on color characteristics of different types of zirconia remains unclear because of limited studies. 9 29 The authors are unaware of a study reported on the comparison of heating rate alteration effects on the color characteristics of various types of multilayer 3Y, 4Y, and 5Y %mol yttria-doped zirconia. Hence, this study aimed to evaluate the influences of zirconia types, layers of zirconia, sintering rate, and their combined effects on color characteristics. The null hypothesis was no statistical significance in color characteristics under different Y contents of zirconia, layers of zirconia, sintering rate, and their combined interactions.

## Materials and Methods

The sample size for this *in vitro* study was estimated using the G*power 3.1 software (Heinrich Heine Universität, Düsseldorf, Germany) according to the statistical data from a previous study 30 using the power of test = 0.9, at 95% level of significance ( *α = 0.05* ) as Equation (1) :



*where Z _α =_ standard normal deviation = 1.96 (α error = 0.05), Z _β =_ standard normal deviation = 1.28 (β error = 0.1), µ _1_ − µ _2_ = mean difference between experimental group =* 0.02, *and σ = standard deviation (σ _1_ =* 0.02, *σ _2_ =* 0.01 *). The number of sample sizes, 15 specimens per group, was used for this experiment* .

## Preparation of the Zirconia Specimens

The multilayer pre-sintered zirconia blanks with different amounts of yttria content, 3Y-TZP, 4Y-PSZ, and 5Y-PSZ (Bloomden Bioceramics, Hunan, China), were sectioned into bar-shaped specimens at an enlarged dimension (width × length × thickness = 14 × 25 × 1.8 mm) to compensate for sintering shrinkage using a precision machine (Mecatome T180, Presi, Eybens, France). The specimens were ground with silicon carbide abrasive paper up to #7000 grit size and subsequently polished with 1 μm diamond suspension in a polisher (Ecomet3, Beuhler, Lake Bluff, Illinois, United States) to achieve a smooth surface. All specimens were cleaned with distilled water to eliminate debris and dried in a desiccator (Ailite GP5, Ailite, Guangdong, China) for 24 hours. The specimens were randomly allocated into 27 groups ( *n* = 15) according to zirconia types, layers (incisal [I], middle [M], cervical [C]), and sintering rate (regular [RS: 10°C/min], fast [FS: 35°C/min], speed [SS: 35°C/min]) ( Table 1 ). The sintering process was performed in the sintering furnace (inFire HTC, Dentsply Sirona, Bensheim, Germany) at the assigned sintering rate until reaching a sintering temperature of 1,530°C with 120 minutes of holding time, and cooled at −10°C/min of cooling rate. Once completed sintering, the specimen was measured with a measuring device (Mitutoyo, Tokyo, Japan) to derive the final dimension (width × length × thickness = 11.2 × 20 × 1.5 mm).

**Table 1:** Zirconia, brand/manufacturers, composition (wt%), and batch number of multilayer (incisal [I], middle [M], cervical [C] layer) 3 mol% yttria-stabilized tetragonal zirconia polycrystal (3Y-TZP), and 4 and 5 mol% yttria partially stabilized zirconia (4Y-PSZ, 5Y-PSZ) sintered with regular (RS; 10°C/min), fast (FS; 10°C/min), and speed (SS; 10°C/min) heat rates (abbreviation of groups [Abv.])

Zirconia					Region	Rate	Group
Type	Brand/Manufacturer	Composition	Batch No.	Abv.			
3 mol% yttria-stabilized tetragonal zirconia polycrystal (3Y-TZP)	Bloomden ST Mu, Bloomden Bioceramics, Hunan, China	≥99% ZrO _2_ + HfO _2_ + Y _2_ O _3_ , 4.5–6% Y _2_ O _3_ , <0.15% Al _2_ O _3_ <0.15% other oxides	S2519112441975	3Y	I	RS	3YIRS
				3Y	I	FS	3YMSF
				3Y	I	SS	3YCSS
3 mol% yttria-stabilized tetragonal zirconia polycrystal (3Y-TZP)	Bloomden ST Mu, Bloomden Bioceramics, Hunan, China	≥99% ZrO _2_ + HfO _2_ + Y _2_ O _3_ , 4.5–6% Y _2_ O _3_ , <0.15% Al _2_ O _3_ <0.15% other oxides	S2519112441975	3Y	M	RS	3YIRS
				3Y	M	FS	3YMFS
				3Y	M	SS	3YCSS
3 mol% yttria-stabilized tetragonal zirconia polycrystal (3Y-TZP)	Bloomden ST Mu, Bloomden Bioceramics, Hunan, China	≥99% ZrO _2_ + HfO _2_ + Y _2_ O _3_ , 4.5–6% Y _2_ O _3_ , <0.15% Al _2_ O _3_ <0.15% other oxides	S2519112441975	3Y	C	RS	3YIRS
				3Y	C	FS	3YMFS
				3Y	C	SS	3YCSS
4 mol% yttria partially stabilized zirconia (4Y-PSZ)	Bloomden ST-Plus Mu, Bloomden Bioceramics, Hunan, China	≥99% ZrO _2_ + HfO _2_ + Y _2_ O _3_ , 7–7.8% Y _2_ O _3_ , <0.15% Al _2_ O _3_ <0.15% other oxides	S2519060831915	4Y	I	RS	4YIRS
				4Y	I	FS	4YMSF
				4Y	I	SS	4YCSS
4 mol% yttria partially stabilized zirconia (4Y-PSZ)	Bloomden ST-Plus Mu, Bloomden Bioceramics, Hunan, China	≥99% ZrO _2_ + HfO _2_ + Y _2_ O _3_ , 7–7.8% Y _2_ O _3_ , <0.15% Al _2_ O _3_ <0.15% other oxides	S2519060831915	4Y	M	RS	4YIRS
				4Y	M	FS	4YMFS
				4Y	M	SS	4YCSS
4 mol% yttria partially stabilized zirconia (4Y-PSZ)	Bloomden ST-Plus Mu, Bloomden Bioceramics, Hunan, China	≥99% ZrO _2_ + HfO _2_ + Y _2_ O _3_ , 7–7.8% Y _2_ O _3_ , <0.15% Al _2_ O _3_ <0.15% other oxides	S2519060831915	4Y	C	RS	4YIRS
				4Y	C	FS	4YMFS
				4Y	C	SS	4YCSS
5 mol% yttria partially stabilized zirconia (5-PSZ)	Bloomden UT Mu, Bloomden Bioceramics, Hunan, China	≥99% ZrO _2_ + HfO _2_ + Y _2_ O _3_ , 9–10% Y _2_ O _3_ , <0.05% Al _2_ O _3_ <0.05% other oxides	S2619111241975	5Y	I	RS	5YIRS
				5Y	I	FS	5YMFS
				5Y	I	SS	5YCSS
5 mol% yttria partially stabilized zirconia (5-PSZ)	Bloomden UT Mu, Bloomden Bioceramics, Hunan, China	≥99% ZrO _2_ + HfO _2_ + Y _2_ O _3_ , 9–10% Y _2_ O _3_ , <0.05% Al _2_ O _3_ <0.05% other oxides	S2619111241975	5Y	M	RS	5YIRS
				5Y	M	FS	5YMFS
				5Y	M	SS	5YCSS
5 mol% yttria partially stabilized zirconia (5-PSZ)	Bloomden UT Mu, Bloomden Bioceramics, Hunan, China	≥99% ZrO _2_ + HfO _2_ + Y _2_ O _3_ , 9–10% Y _2_ O _3_ , <0.05% Al _2_ O _3_ <0.05% other oxides	S2619111241975	5Y	C	RS	5YIRS
				5Y	C	FS	5YMFS
				5Y	C	SS	5YCSS

## Determination of the Color Characteristics

The color characteristics of multilayer zirconia specimens with different sintering rates were achieved using a spectrophotometer (ColorQuest XE, Hunter, Reston, Virginia, United States). The machine was set at a 10-degree observer angle, 100% UV, D-65 illuminant at the standard wavelength between 380 and 780 nm, and 4 mm diameter of the aperture. It was calibrated with a standard white tile before starting the measurements. A transparent acrylic template was employed to maintain the position of the specimen during the optical parameter measurement. The incisal (I), middle (M), and cervical (C) layers of specimens were measured independently at the middle of three portions (left, central, and right) for each layer. The Commission International de I'Eclairage (CIE L*a*b*) color space system was used to determine L*, a*, and b* color parameters, which were attained for the lightness, the red-green coordinate, and the yellow-blue coordinate of specimens, respectively, against the white (W) (L* _W_ = 96.70, a* _W_ = 0.10, b* _W_ = 0.20) and black (B) (L* _W_ = 30.53, a* _W_ = 0.95, b* _W_ = 0.36) background. Then, the CIEDE2000 was used to determine for translucency parameter (TP _00_ ), CR, OP, and color difference (∆E _00_ ). The relative translucency was determined from the TP _00_ values that were calculated from the differences between color determinants on black and white backgrounds, according to Equation 2 :



*where L′, C′, and H′ represent the differences in lightness, chroma, and hue of the specimens against black (B) and white (W) backgrounds; R _T_ is the rotational function that accounts for the interaction between chroma and hue difference in the blue region; S _L_ , S _C_ , and S _H_ are the weighting functions for lightness, chroma, and hue; and K _L_ , K _C_ , and K _H_ are the correct terms for experimental conditions, which were set at 1 in the present study.*

The contrast was determined from the CR values using Equations 3 and 4 , which ranged from 0.0 (transparent) to 1.0 (perfectly opaque). In the tristimulus color space, Y represents the brightness illuminance; Y _B_ and Y _W_ are the values of a specimen placed on the black and white backgrounds, respectively; and Y _*n*_ is equal to 100.





The opalescence was determined from the OP values that were achieved by using Equation (5) .



The color difference was determined from the ∆E _00_ , which was calculated from the data set of each specimen on a standard white background, compared to the mean coordinate of the same type and layer of zirconia that were sintered under regular sintering rate, as shown in Equation 6 :



*where L′, C′, and H represent the differences in the lightness, chroma, and hue of a set of samples.*

## Determination of the Microstructure and Chemical Composition

Three specimens represented as sintered surfaces were randomly selected from each group for microscopic examination. The specimens were cleaned with distilled water, dried in the auto-desiccator at normal ambient temperature for 24 hours, and then coated with gold-palladium in a sputter coater (K500X, Quorum Technology, Kent, United Kingdom) at 10 mA current, 130 mTorr vacuum, for 3 minutes. The specimen surfaces were examined for grain morphology and grain size with a scanning electron microscope (SEM; SU3800, Hitachi, Tokyo, Japan) and grain size analyzer program (GSA program, KKU, Khon Kaen, Thailand) at ×10K magnification. The chemical compositions were characterized with energy dispersive spectroscopy (EDS; Oxford, High Wycombe, United Kingdom).

## Determination of the Phase Composition

The fraction of c-, t-, and m-phases of all sample groups was observed using X-ray diffraction (XRD; Bruker, Karlsruhe, Germany). The zirconia specimens' surface was inspected using Cu k-α radiation at a diffraction angle (2 *θ* ) of 20 to 90 degrees, with a step size of 0.02° for a second interval. The XRD patterns were generated using Origin-Pro 2019 (OriginLab, Wellesley, Massachusetts, United States) to analyze the relative proportions of phases based on the peak intensity using the Match-3.0 software (Crystal Impact, Bonn, Germany). The peaks were cross-referenced to the Joint Committee of Powder Diffraction Standards database files (PDFs) No. 03-0640, 02-0733, and 07-0343, for c-, t-, and m-phases, respectively. The relative intensities of peaks for m-phase (I _m_ ), t-phase (I _t_ ), and c-phase (I _c_ ) were analyzed by the X'Pert–Plus software (Philips, Almelo, The Netherlands). The calculation was performed upon matching a Pseudo-Voigt distribution to the courtesy peak and considering the area beneath the curve. Considering the influence of yttria on the lattice parameters, the corrected factor of 1.311 was used to calibrate the nonlinear curve of assimilated intensity ratios against volume fraction. The Garvie–Nicholson formula was applied for calculating the proportion of m-phase ( *X*_m_ ), t-phase ( *X*_t_ ), and c-phase ( *X*_c_ ) as Equations 7 , 8 , and 9 .







## Determination of the Surface Topography

The surface topography and surface roughness of the zirconia specimens were examined with a three-dimensional (3D) digital microscope (Olympus DSX1000, Evident, Tokyo, Japan), and further analyzed with the image analysis software (PRECiV-Olympus, Evident). The brightfield mode at ×79 magnification with high contrast and high dynamic range texture was selected to evaluate the 3D topography at the area of 3.6 × 3.6 mm ^2^ , for five areas of specimen in each group, and calculated the average surface roughness (Ra).

### Statistical Analysis

The data were executed with the Shapiro–Wilk test for normality test, and Levene's test for homoscedasticity test using statistical software (IBM SPSS V-26, SPSS, Chicago, Illinois, United States). Since the data were normally distributed and presented homoscedasticity ( *p* > 0.05), the three-way ANOVA and post-hoc Bonferroni multiple comparisons were performed to detect substantial variations in color characteristics (TP _00_ , CR, OP, and ∆E _00_ ) of the 3, 4, and 5 mol% yttria-containing multilayer zirconia, under different layers and different sintering rates. A statistically significant difference was set at *p <* 0.05. Descriptive analysis was employed to assess the grain size, elemental composition, relative phase composition, and surface roughness of the zirconia.

## Results

**Fig. 1:**
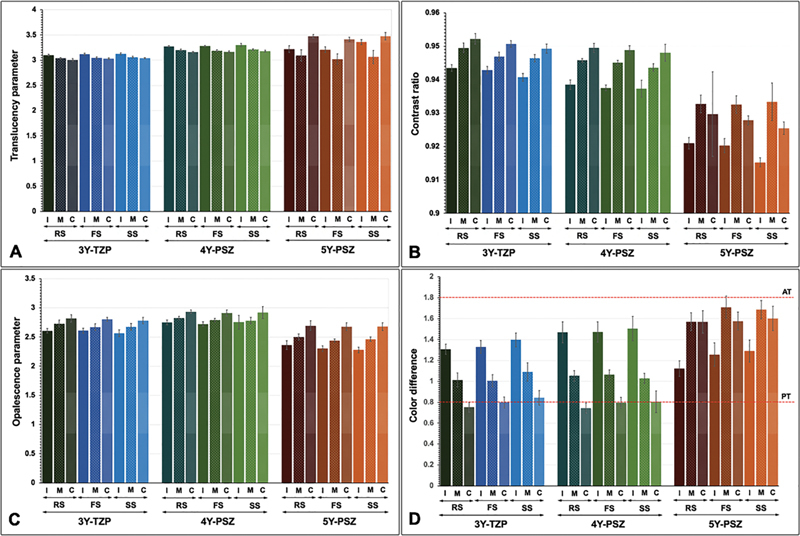
Translucency parameter ( **A** ), contrast ratio ( **B** ), opalescence parameter ( **C** ), and color difference ( **D** ) within perceptible threshold (PT) and acceptable threshold (AT) of multilayer (incisal [I], middle [M], cervical [C] layer) 3Y-TZP, 4Y-PSZ, and 5Y-PSZ upon sintered at regular (RS), fast (FS), and speed (SS) sintering rates.

**Fig. 2:**
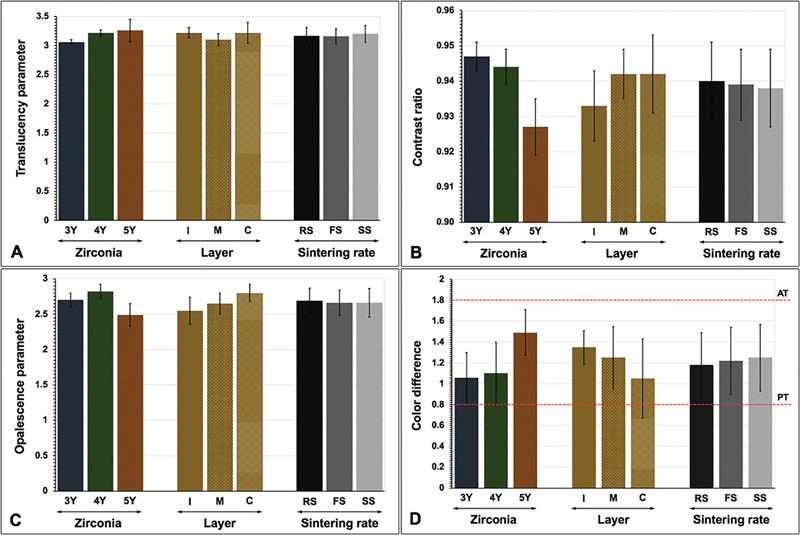
Influence of type of zirconia (3Y-TZP, 4Y-PSZ, and 5Y-PSZ), location of multilayer (incisal [I], middle [M], cervical [C] layer), and sintering rate (regular [RS], fast [FS], and speed [SS]) on translucency parameter ( **A** ), contrast ratio ( **B** ), opalescence parameter ( **C** ), and color difference ( **D** ) within perceptible (PT) and acceptable thresholds (AT).

The mean and standard deviation of TP _00_ , CR, OP, ∆E _00_ , Ra, grain size distribution, relative phase content, and chemical elements of multilayer fully stabilized zirconia containing 3Y, 4Y, and 5Y, in I, M, and C layers, under RS, FS, and SS sintering rates were reported ( Table 2 and Figs. 1 2 3 ). Three-way ANOVA indicated that the color parameters, including TP _00_ , CR, OP, and ΔE _00_ , were significantly influenced by zirconia type, layers of zirconia, and sintering rate ( *p <* 0.05), except the interaction of zirconia type and sintering rate for CR and OP, the interaction of layer of zirconia and sintering rate for CR, OP, and ΔE _00_ , and three-factor interaction for OP ( *p > 0.05* ) as presented in Table 3 . Post-hoc Bonferroni multiple comparisons indicated that types of zirconia, layer, and sintering rate presented a statistically significant effect on TP _00_ , CR, OP, and ΔE _00_ ( *p <* 0.05), except for groups of I/C and RS/FS in TP _00_ , M/C in CR, and FS/SS in OP ( Table 4 and Fig. 2 ). Considering type of zirconia, the study suggested that increasing the amount of yttria content in zirconia significantly increased TP _00_ and ΔE _00_ , but significantly decreased CR and OP ( *p <* 0.05; Table 4 and Fig. 2 ). Reflecting the layer of zirconia, the statistics indicated statistically significant higher TP _00_ and ΔE _00_ , but significantly lowered CR and OP for I than M and C layers ( *p <* 0.05). Nevertheless, there was no significant difference in TP _00_ between the I and C layers, as well as no significant difference in CR between the M and C layers ( *p >* 0.05; Table 4 and Fig. 2 ). Regarding sintering rate, the study indicated that sintering zirconia with SS resulted in a significant increase in TP _00_ and ΔE _00_ , but significant reduction in CR and OP compared to FS and RS ( *p <* 0.05). Yet, there was no significant difference in TP _00_ between RS and FS, and no significant difference in OP between SS and FS ( *p >* 0.05; Table 4 and Fig. 2 ).

**Table 2:** Mean, standard deviation (SD) of translucency parameter (TP _00_ ), contrast ratio (CR), opalescence parameter (OP), color difference (∆E _00_ ), surface roughness (Ra), percentage of fine (f), medium (m), and large (l) grain size distribution (%), and relative cubic (c-), tetragonal (t-), and monoclinic (m-) phase content (wt.%) of multilayer (incisal [I], middle [M], cervical [C] layer) 3Y-TZP, 4Y-PSZ, and 5Y-PSZ upon sintered at regular (RS), fast (FS), and speed (SS) sintering rates

Group			*n*	TP _00_	CR	OP	∆E _00_	Ra	Grain size (%)	Phase (wt.%)
Zirconia	Layer	Rate		Mean ± SD	Mean ± SD	Mean ± SD	Mean ± SD	Mean ± SD	f/m/l	*c* -/ *t* -/ *m* -
3Y	I	RS	15	3.10 ± 0.02	0.94 ± 0.01	2.61 ± 0.04	1.31 ± 0.05	4.45 ± 1.14	98.4/1.6/0.0	18.0/74.4/7.6
3Y	M	RS	15	3.04 ± 0.02	0.95 ± 0.001	2.73 ± 0.06	1.01 ± 0.07	4.19 ± 1.39	97.7/2.3 /0.0	18.0/74.4/7.6
3Y	C	RS	15	3.01 ± 0.02	0.95 ± 0.02	2.82 ± 0.06	0.75 ± 0.05	3.17 ± 1.27	98.6/1.4/0.0	18.0/74.4/7.6
3Y	I	FS	15	3.12 ± 0.02	0.94 ± 0.01	2.61 ± 0.04	1.33 ± 0.06	5.13 ± 2.38	97.7/2.3/0.0	17.4/76.9/5.7
3Y	M	FS	15	3.05 ± 0.02	0.95 ± 0.01	2.67 ± 0.06	1.01 ± 0.06	3.99 ± 1.21	97.1/2.9/0.0	17.4/76.9/5.7
3Y	C	FS	15	3.03 ± 0.01	0.95 ± 0.01	2.80 ± 0.03	0.80 ± 0.05	4.04 ± 1.04	96.5/3.5/0.0	17.4/76.9/5.7
3Y	I	SS	15	3.13 ± 0.02	0.94 ± 0.01	2.56 ± 0.06	1.40 ± 0.07	4.02 ± 1.15	97.0/3.0/0.0	17.4/75.7/6.9
3Y	M	SS	15	3.06 ± 0.02	0.95 ± 0.01	2.68 ± 0.06	1.09 ± 0.09	4.04 ± 1.28	96.8/3.2/0.0	17.4/75.7/6.9
3Y	C	SS	15	3.04 ± 0.01	0.95 ± 0.01	2.78 ± 0.05	0.84 ± 0.07	4.67 ± 1.51	98.2/1.8/0.0	17.4/75.7/6.9
4Y	I	RS	15	3.27 ± 0.01	0.94 ± 0.01	2.75 ± 0.04	1.47 ± 0.10	3.44 ± 0.90	82.8/17.2/0.0	28.7/70.8/0.1
4Y	M	RS	15	3.20 ± 0.02	0.95 ± 0.01	2.82 ± 0.03	1.05 ± 0.05	4.06 ± 1.02	72.7/27.3/0.0	28.7/70.8/0.1
4Y	C	RS	15	3.16 ± 0.01	0.95 ± 0.01	2.93 ± 0.03	0.74 ± 0.05	3.37 ± 0.96	74.1/25.1/0.8	28.7/70.8/0.1
4Y	I	FS	15	3.28 ± 0.01	0.94 ± 0.01	2.72 ± 0.04	1.47 ± 0.10	3.76 ± 1.09	75.2/24.8/0.0	29.5/70.2/0.3
4Y	M	FS	15	3.19 ± 0.02	0.94 ± 0.01	2.79 ± 0.03	1.06 ± 0.05	4.48 ± 0.81	71.6/28.4 /0.0	29.5/70.2/0.3
4Y	C	FS	15	3.16 ± 0.02	0.95 ± 0.01	2.91 ± 0.05	0.80 ± 0.05	2.92 ± 0.68	76.0 /23.6/0.4	29.5/70.2/0.3
4Y	I	SS	15	3.30 ± 0.03	0.94 ± 0.02	2.75 ± 0.12	1.50 ± 0.12	2.92 ± 0.76	79.5/20.5/0.0	30.4/69.4/0.2
4Y	M	SS	15	3.21 ± 0.02	0.94 ± 0.01	2.78 ± 0.05	1.03 ± 0.05	3.20 ± 1.64	74.5/25.5/0.0	30.4/69.4/0.2
4Y	C	SS	15	3.18 ± 0.02	0.95 ± 0.03	2.92 ± 0.10	0.81 ± 0.10	4.26 ± 1.25	76.3/23.3/0.4	30.4/69.4/0.2
5Y	I	RS	15	3.22 ± 0.07	0.92 ± 0.02	2.36 ± 0.08	1.12 ± 0.07	2.95 ± 0.94	34.5/51.7/13.8	51.5/48.3/0.2
5Y	M	RS	15	3.09 ± 0.11	0.93 ± 0.03	2.50 ± 0.06	1.57 ± 0.09	3.28 ± 0.68	44.6/41.3/14.1	51.5/48.3/0.2
5Y	C	RS	15	3.47 ± 0.04	0.93 ± 0.03	2.69 ± 0.09	1.57 ± 0.11	3.77 ± 0.72	29.4/57.7/12.9	51.5/48.3/0.2
5Y	I	FS	15	3.21 ± 0.06	0.92 ± 0.02	2.30 ± 0.05	1.26 ± 0.11	3.00 ± 0.85	46.4/43.3/10.3	52.8/46.6/0.6
5Y	M	FS	15	3.02 ± 0.10	0.93 ± 0.03	2.44 ± 0.03	1.71 ± 0.11	3.51 ± 1.04	37.5/45.0/17.5	52.8/46.6/0.6
5Y	C	FS	15	3.41 ± 0.04	0.93 ± 0.01	2.68 ± 0.07	1.58 ± 0.09	4.12 ± 1.24	46.6/39.8/13.6	52.8/46.6/0.6
5Y	I	SS	15	3.36 ± 0.05	0.92 ± 0.01	2.28 ± 0.05	1.29 ± 0.10	3.19 ± 0.79	40.6/50.5/8.9	55.4/44.3/0.4
5Y	M	SS	15	3.06 ± 0.13	0.93 ± 0.06	2.46 ± 0.04	1.69 ± 0.09	3.25 ± 0.80	31.9/54.3/13.8	55.4/44.3/0.4
5Y	C	SS	15	3.47 ± 0.07	0.92 ± 0.02	2.68 ± 0.07	1.60 ± 0.12	2.89 ± 0.63	32.2/54.4/13.3	55.4/44.3/0.4

**Fig. 3:**
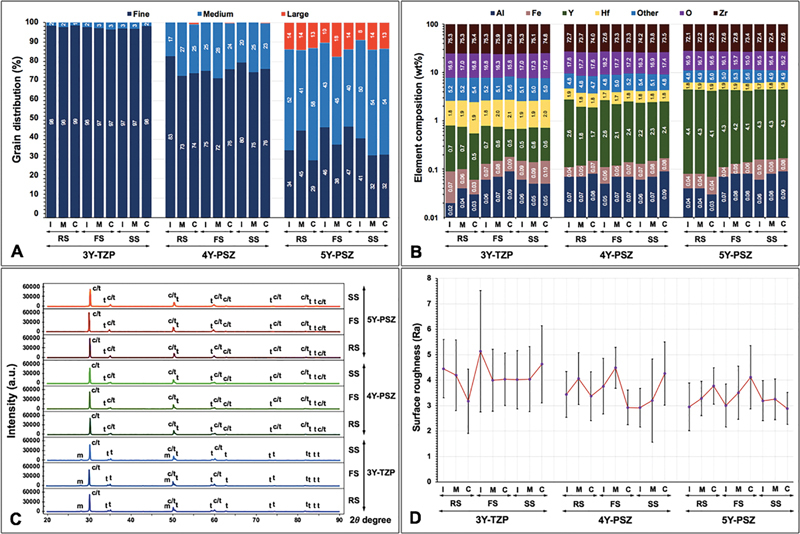
( **A** ) Grain distribution, ( **B** ) elemental composition, ( **C** ) X-ray diffraction pattern, and ( **D** ) surface roughness of multilayer (incisal [I], middle [M], cervical [C] layer) 3Y-TZP, 4Y-PSZ, and 5Y-PSZ upon sintered at regular (RS), fast (FS), and speed (SS) sintering rates.

**Table 3:** Three-way ANOVA of (a) translucency parameter (TP _00_ ), (b) contrast ratio (CR), (c) opalescence parameter (OP), and (d) color difference (∆E _00_ ) of multilayer (incisal [I], middle [M], cervical [C] layer) 3Y-TZP, 4Y-PSZ, and 5Y-PSZ upon sintered at regular (RS), fast (FS), and speed (SS) sintering rates

(a) ANOVA of TP _00_ upon different factors					
Source	SS	df	MS	F	*p*
Zirconia types	2.864	2	1.432	594.691	0.001
Layers	1.211	2	0.605	251.414	0.001
Sintering rates	0.110	2	0.055	22.791	0.001
Zirconia types * layers	2.798	4	0.699	290.478	0.001
Zirconia types * sintering rates	0.085	4	0.021	8.875	0.001
Layers * sintering rates	0.054	4	0.013	5.559	0.001
Zirconia types * layers * sintering rates	0.084	8	0.010	4.345	0.001
Error	0.910	378	0.002		
**(b) ANOVA of CR upon different factors**					
Zirconia type	0.033	2	0.016	1705.246	0.001
Layers	0.007	2	0.004	387.582	0.001
Sintering rate	0.000	2	0.000	22.725	0.001
Zirconia type * layers	0.001	4	0.000	38.739	0.001
Zirconia type * sintering rate	4.054	4	1.014	1.062	0.375
Layers * sintering rate	5.860	4	1.465	1.536	0.191
Zirconia type * layers * sintering rate	0.000	8	2.145	2.249	0.023
Error	0.004	378	9.541		
**(c) ANOVA of OP upon different factors**					
Zirconia types	7.613	2	3.807	1108.221	0.001
Layers	4.321	2	2.160	628.931	0.001
Sintering rates	0.104	2	0.052	15.110	0.001
Zirconia types * layers	0.487	4	0.122	35.436	0.001
Zirconia types * sintering rates	0.019	4	0.005	1.385	0.239
Layers * sintering rates	0.016	4	0.004	1.150	0.332
Zirconia types * layers * sintering rates	0.036	8	0.004	1.298	0.243
Error	1.298	378	0.003		
** (d) ANOVA ∆E _00_ upon different factors **					
Zirconia types	14.890	2	7.445	1124.918	0.001
Layers	6.080	2	3.040	459.320	0.001
Sintering rates	0.355	2	0.178	26.821	0.001
Zirconia types * layers	16.735	4	4.184	632.173	0.001
Zirconia types * sintering rates	0.139	4	0.035	5.232	0.001
Layers * sintering rates	0.026	4	0.006	0.968	0.425
Zirconia types * layers * sintering rates	0.137	8	0.017	2.596	0.009
Error	2.502	378	0.007		

Abbreviations: df, degree of freedom; F, F-ratio; MS, mean square; NB: SS, sum of squares.

**Table 4:** Post-hoc Bonferroni multiple comparisons of (a) translucency parameter (TP _00_ ), (b) contrast ratio (CR), (c) opalescence parameter (OP), and (d) color difference (∆E _00_ ) of multilayer (incisal [I], middle [M], cervical [C] layer] 3Y-TZP, 4Y-PSZ, and 5Y-PSZ upon sintered at regular (RS), fast (FS), and speed (SS) sintering rates

** (a) Post-hoc multiple comparison of TP _00_ as a function of zirconia, region, and sintering rate **											
**Zirconia**	**3Y**	**4Y**	**5Y**	**Layer**	**I**	**M**	**C**	**Sintering rate**	**RS**	**FS**	**SS**
**3Y**	1	0.001	0.001	**I**	1	0.001	1	**RS**	1	0.237	0.001
**4Y**		1	0.001	**M**		1	0.001	**FS**		1	0.001
**5Y**			1	**C**			1	**SS**			1
**(b) Post-hoc multiple comparison of CR as a function of zirconia, region, and sintering rate**											
**Zirconia**	**3Y**	**4Y**	**5Y**	**Layer**	**I**	**M**	**C**	**Sintering rate**	**RS**	**FS**	**SS**
**3Y**	1	0.001	0.001	**I**	1	0.001	0.001	**RS**	1	0.013	0.001
**4Y**		1	0.001	**M**		1	0.405	**FS**		1	0.001
**5Y**			1	**C**			1	**SS**			1
**(c) Post-hoc multiple comparison of OP as a function of zirconia, region, and sintering rate**											
**Zirconia**	**3Y**	**4Y**	**5Y**	**Layer**	**I**	**I**	**M**	**Sintering rate**	**RS**	**FS**	**SS**
**3Y**	1	0.001	0.001	**I**	1	0.001	0.001	RS	1	0.001	0.001
**4Y**		1	0.001	**M**		1	0.001	FS		1	1
**5Y**			1	**C**			1	SS			1
**(d) Post-hoc multiple comparison of** ∆ ** E _00_ as a function of zirconia, region, and sintering rate **											
**Zirconia**	**3Y**	**4Y**	**5Y**	**Layer**	**I**	**M**	**C**	**Sintering rate**	**RS**	**FS**	**SS**
**3Y**	1	0.001	0.001	**I**	1	0.001	0.001	**RS**	1	0.001	0.001
**4Y**		1	0.001	**M**		1	0.001	**FS**		1	0.031
**5Y**			1	**C**			1	**SS**			1

Concerning the color alteration, the study indicated that 5Y revealed a significantly higher color alteration than 4Y and 3Y, respectively, suggesting that increasing Y content in zirconia exhibited a significantly easier color alteration ( *p <* 0.05; Table 4 and Fig. 2D ). Furthermore, the study discovered that the color alteration in the I layer was significantly higher than those in M and C layers ( *p <* 0.05; Table 4 and Fig. 2D ). This study indicated that the sintering at the SS sintering rate produced a significantly higher color alteration than sintering at FS and RS for all types of zirconia ( *p <* 0.05; Table 4 and Fig. 2D ). Nevertheless, color alterations for all groups were between PT (ΔE _00_ ≤ 0.8) and AT (ΔE _00_ ≤ 1.8) ( Fig. 1 ). Color alteration of zirconia under different types of zirconia (3Y, 4Y, and 5Y), different layers (I, M, and C), and different sintering rates (RS, FS, and SS) was within the AT ( Fig. 2D ).

**Fig. 4:**
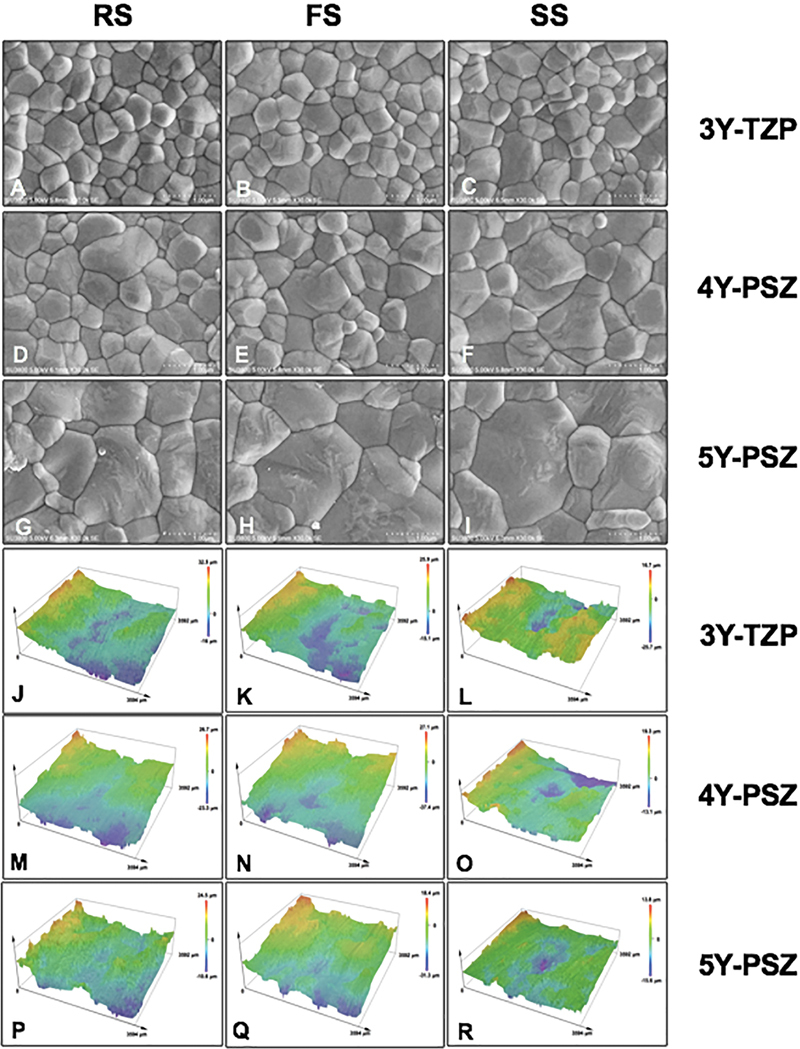
Scanning electron microscopy photomicrographs at ×30K magnification indicated grain size and grain distribution ( **A** – **I** ), and surface topography indicated surface roughness ( **J** – **R** ) of multilayer 3Y-TZP (A–C; J–L), 4Y-PSZ (D–F; M–O), and 5Y-PSZ (G–I; P–R) upon sintered at regular (RS; A, D, G, J, M, and P), fast (FS; B, E, H, K, N, and Q), and speed (SS; C, F, I, L, O, and R) sintering rates.

The SEM photomicrographs at ×10K magnification were quantified for percentage (%) grain size distribution, categorized as fine grains (F, 0.01–0.99 µm), medium grains (M, 1.00–1.99 µm), and large grains (L, 2.00–2.99 µm) ( Table 2 and Fig. 3A ). All 3Y groups demonstrated mainly F grain and a small amount of M grain. All 4Y groups principally composed of F grain and a minor amount of M grain. However, higher percentages of M grain in 4Y than 5Y groups were indicated. Both 3Y and 4Y groups were rarely composed of the L grain. All 5Y groups were composed of M grains more than F and L grains. The grain size distribution in the same type of zirconia was not influenced by the layer of zirconia and sintering rate. Notably, 3Y demonstrated the densely packed F grain size with a mostly round-shape appearance, whereas 4Y presented a mixture of F grains with a round shape, circumferentially arranged around M grains with a polygonal shape appearance, while 5Y mostly presented densely compacted L grains with a polygonal shape appearance ( Fig. 4A–I ). The chemical composition (wt.%) of all groups of zirconia consisted of zirconia (Zr) and oxygen (O) as principal elements. Besides, yttria (Y), aluminum (Al), osmium (OS), hafnium (Hf), thorium (Th), and ferrous (Fe) were minor elements. The Y element varied according to the type of zirconia. The 5Y groups exhibited the highest Y content (4.2–4.7%), whereas the 3Y groups exhibited the lowest Y content (0.6–0.8%) ( Table 2 and Fig. 3B ).

The XRD pattern for all zirconia groups was composed of *c* -, *t* -, and *m* -phases ( Table 2 and Fig. 3C ). The main peak of the *c* -phase was located at the diffraction angle (2 *θ* ) of 30.168°, and belonged to the *111* crystalline plane, while the minor peaks were found at 2 *θ* of 35.023°, 50.375°, 60.026°, and 74.320°, respectively, matched with *200, 220, 311* , and 400 planes. The principal peak of the *t* -phase was noticed at 2 *θ* of 30.484°, paired with the *111* plane, whilst the minor peaks were found at 2 *θ* of 35.597°, 50.978°, and 60.459°, correspondingly fitted with *002* , *220,* and *004* planes. The chief peak of the *m* -phase was identified at 2 *θ* of 28.218°, which belonged to the (− *1)11* plane, while the minor peaks were found at 2 *θ* of 31.475°, 34.196°, and 50.167°, singly paired with the *111* , *002* , and *220* planes. The multiplicity of *c* -, *t* -, and *m* -phase varied with zirconia types and sintering rate. The 3Y and 4Y zirconia composed mainly of the *t* -phase (74.4–78.2% and 62.5–70.8%), while 5Y zirconia encompassed principally of the *c* -phase (52.8-58.0%). The *m* -phase was the most diminutive phase content in all groups. The sintering rate affected the relative phase contents by increasing the c-phase upon raising the sintering rate in 4Y and 5Y zirconia.

The surface roughness revealed the highest Ra in the 3YIFS group (5.13 ± 2.38 µm), and the lowest Ra in the 5YCSS group (2.89 ± 0.62 µm). The Ra ranged between 5.13 ± 2.38 and 3.17 ± 1.27 µm for 3Y, between 4.48 ± 0.81 and 2.92 ± 0.77 µm for 4Y, and between 4.12 ± 1.24 and 2.89 ± 0.63 µm for 5Y ( Table 2 and Fig. 3D ). The 3D surface topography of 3Y, 4Y, and 5Y zirconia under varied sintering rates is shown in Fig. 4J–R . The color gradience represented the level of surface roughness, in which the higher areas were demonstrated in red-orange-yellow gradience, whereas the lower areas were demonstrated in green-blue-purple gradience. The Ra tended to decrease from 3Y to 4Y and 5Y zirconia. The layers and the sintering rate did not exhibit any influence on Ra.

## Discussion

A comprehensive consideration of color characteristics including translucency, contrast, opalescence, and color alteration is an essential determinant for predicting an aesthetic ceramic restorative material to reproduce the natural appearance of dentition in clinical practice. 1 2 This study intended to improve the color characteristics of 3Y, 4Y, and 5Y multilayer zirconia through the sintering process by varying sintering rates. The study revealed statistically significant effects of zirconia types, zirconia layers, and sintering rates, as well as their interactions, on all color characteristics, except the interaction of zirconia type and sintering rate for CR and OP, the interaction of layer of zirconia and sintering rate for CR, OP, and ΔE _00_ , and three-factor interaction for OP. Therefore, the null hypotheses were partially rejected. The study signified that translucency, contrast, opalescence, and color alteration of the monolithic multilayer zirconia were influenced by zirconia types, layers of zirconia, and sintering rates. This is possibly related to the microstructure of the zirconia as supported by other studies. 4 5

Considering translucency, the study revealed that the translucency increased as the grain size of zirconia enlarged. The amount of large zirconia grains was probably associated with translucency since they promoted light transmission, reducing the scattering effect of the incidence light at the grain boundaries as supported by other studies. 4 5 This experiment revealed that the 5Y containing zirconia exhibited higher translucency than 4Y and 3Y. This is possibly associated with the increased amount of Y content, which could enhance the growth of zirconia grain as evidenced by the SEM photomicrographs and EDS analysis. Likewise, the increasing amount of Y in zirconia may well escalate the c-phase as indicated by XRD. The isotropic structure of the c-phase at the grain boundary may perhaps improve the light transmission, and diminish the light reflection and deflection through the monolithic zirconia. 5 11 13 This investigation also indicated that the less the surface roughness, the higher the translucency of zirconia, as evidenced by higher Ra of 3Y than 4Y and 5Y. In addition, the higher the amount of Y included in zirconia, the larger the grain size exhibited, which is inversely related to the Ra value. The smooth surface probably promotes translucency by reducing the effect of the surface on the light reflection and deflection. 10 The differences in translucency among the layers of zirconia were indicated in this study. This finding is related to the different concentrations of the color pigment among zirconia layers that are added in the manufacturing process. The middle layer was the transition area of the color gradient that might have inconsistent color pigment distribution, which probably led to more light scattering effect, which evoked the lower translucency in the middle portion as supported by other studies. 5 6 This study also indicated that the sintering rate affected the translucency of zirconia. The translucency could be promoted by speeding the sintering rate as described in the SS strategy. This is possibly correlated to the grain size enlargement upon SS sintering rate. The rapid rise in temperature in the SS protocol possibly steered the simultaneous speedy growth of zirconia grains, which feasibly promoted surface integration of the grain boundaries. Consequently, this incidence could lead to pore size dwindling via the zirconia grain condensation process. 16 26 Therefore, enhancing the translucency of the zirconia through the SS sintering strategy is possibly caused by the grain densification and the pore reduction process that improves the light transmission throughout the zirconia. The influence of the speed sintering rate on the translucency of zirconia was supported by other studies. 3 24 27 Nonetheless, this phenomenon, as opposed to other studies, possibly correlates to the use of different zirconia brands and different sintering protocols. 23 25

Concerning opalescence, the restorative material should possess an OP value close to the OP of human teeth to imitate tooth appearance. Significant differences exist among zirconia type, layers of zirconia, and sintering rate in their effects on OP. This finding is perhaps associated with the variation of the pigment substances and the additive chemical compositions that comprise the zirconia blank during the manufacturing process, which causes the light reflection or refraction from these substances. The 3Y and 4Y possessed significantly higher OP than 5Y, which is perhaps related to the high scattering effect of the t-phase that presented as the main phase composition for 3Y and 4Y, compared to 5Y. Significant differences in OP among layers for each type of zirconia were indicated. The OP values respectively increased from I to M to C layer. This phenomenon is probably related to the coloring pigments added for increasing chroma and value in each layer, which affected the light path and resulted in different occurrences of opalescence, which was well coincided with previous studies. 18 20 Furthermore, the study found that the opalescence significantly decreased when increasing the sintering rate. The finding is possibly associated with the microstructure of the zirconia grains that correlated with the effect of light transmittance and reflectance at the grain boundaries. The high transmission of light (high translucency) is associated with low opalescence due to the low light scattering at the grain boundaries and pores between the zirconia grains, as supported by a previous study. 28 The OP value observed in the present study (2.28–2.93) was lower than the OP value of the human enamel (19.8–27.6); however, the OP in this study was within the normal range of the contemporary dental ceramic (1.6–21.6). 19 28

The color difference value is crucial for determining the amount of color alteration of different types of zirconia upon varying sintering rates, compared to the regular sintering rate, with a lower ΔE _00_ value indicating less color alteration or better color stability. The study indicated a significant influence of the zirconia types, layers of zirconia, and sintering rates on the color difference. Among the zirconia types, the 3Y had the lowest ΔE _00_ value, indicating the least color alteration. Considering layers of zirconia, the I layer exhibited a higher color alteration than the M and C layers, respectively. Regarding the sintering rate strategy, the SS rate produced a higher color alteration than FS and RS, respectively. This might be related to the high translucency appearance of zirconia. The high-translucency ceramics were highly sensitive to color mismatching due to the microstructure of the crystalline phases that could reduce the scattering of light. 18 This occurrence was supported by previous studies that reported a strong correlation between translucency and color appearance difference. 9 Moreover, the background color and the thickness of the ceramics also influence ΔE _00_ , especially in the high-translucency ceramics. 17 However, the ΔE _00_ values for all zirconia groups in the present study were within the AT (ΔE _00_ ≤ 1.8), which means clinically acceptable. 7 17 The study corresponded with a previous study that found the speed sintering protocol produced the ΔE _00_ lower than the AT for most zirconia brands (ΔE _00_ = 1.24–1.59). 11

Based on the present study, the color characteristics of monolithic multilayer zirconia were influenced by the zirconia types, layers of zirconia, and sintering rates. This implied that the selection of difference sintering rates especially in the speed sintering could be applied for sintering the 3Y, 4Y, and 5Y multilayer zirconia with minimal effect on color characteristics _._ The SS sintering rate can enhance translucency, but may slightly reduce opalescence, with tiny color alteration within the AT for all types of zirconia. Nevertheless, the study had a distinct limitation since it considered only the influence of the sintering rate on the color characteristics of 3Y, 4Y, and 5Y multilayer zirconia. The effect of varied sintering rates on color characteristics of monochrome 3Y, 4Y, and 5Y zirconia, as well as their effect on the mechanical properties, and the precision of the restoration should be further investigated to fulfill the clinical requirement.

## Conclusion

This study could be summarized that color characteristics were influenced by zirconia types, layers of zirconia, and sintering rate. Alteration in the sintering rate significantly affected translucency, contrast, opalescence, and color appearance of zirconia, depending on the quantities of yttria content and layer of zirconia. However, adjustment of sintering rates to achieve good color characteristics of zirconia with appropriate processing time for chairside restorative treatment was feasible. The speed sintering rate was a more effective method than fast and regular sintering rates to provide an efficient achievement of better translucency, contrast, and opalescence for 5Y more than 4Y and 3Y, and effectively affected incisal more than the middle and cervical layers of zirconia, with clinically acceptable color alteration. Hence, to achieve the most auspicious color characteristics, the speed sintering rate was recommended in the sintering process for all types of yttria containing multilayer zirconia.
